# Atomic force measurements on the specifically orientated membrane protein TGR5 reconstituted inside a tethered bilayer lipid membrane

**DOI:** 10.1186/2047-783X-19-S1-S11

**Published:** 2014-06-19

**Authors:** Anna Bronder, Arpita Roychoudhury, Dieter Häussinger, Filipp Oesterhelt

**Affiliations:** 1 Institute of Physical Biology, AG Nanoanalytics, Heinrich Heine University, 40225 Düsseldorf, Germany; 2 Clinic of Gastroenterology, Hepatology and Infectious Diseases, Heinrich Heine University, 40225 Düsseldorf, Germany

## Background

The G-protein coupled bile acid receptor (GPBAR1), commonly known as TGR5 is a transmembrane protein associated with diabetes, metabolic syndrome, inflammation and cancer in various organs. Together with these diseases a TGR5 deficiency, overexpression or mutation can often be observed. Due to this association of TGR5 and its mutants with different disorders, TGR5 is seen as a potential drug target. For the successful development of TGR5 agonists, it is important to have detailed information on the protein's structure and the structural changes caused by mutations. TGR5 is activated by bile acids (BAs), making BAs potential drug candidates [1]. BAs are signaling molecules with systemic endocrine functions, such as the regulation of bile acid, glucose & lipid metabolism, immune response and cell proliferation and differentiation [2]. However, BAs target several nuclear and plasma receptors, like the farnesol X receptor and TGR5, at once [3]. This makes it difficult to find TGR5 specific agonists. Knowledge about the interactions between BAs and TGR5 could help in the development of BA-derivatives and other new synthetic agonists exclusively targeting TGR5 with high efficiency. To study proteins, especially membrane proteins, different methods, e.g. atomic force microscopy and total internal reflection fluorescence microscopy can be applied. However, these methods are in need of a solid support and the protein being present in a defined orientation. In our group we have studied bacterial membrane proteins, e.g. bacteriorhodopsin, with single-molecule atomic force microscopy and spectroscopy [4,5]. The atomic force microscope (AFM) is a tool to image biological surfaces with sub-nanometer resolution. Important for biomolecules, like transmembrane proteins, is the fact that they can be studied in their natural environment, an aqueous solution. Force measurements performed with the AFM on individual molecules reveal inter- and intramolecular interaction at the pN scale [5], showing structural details, information on protein stability and the interactions between different molecules. These force measurement take advantage of the interaction between AFM tip and protein to pull the protein out of the membrane. When the terminus of the protein adsorbs to the tip, we can observe the unfolding of all protein domains as the tip moves away from the surface. Some domains of membrane proteins are embedded inside the membrane, e.g. α-helices, while other domains, e.g. loops, are outside. Using force measurements the different constitutions and chain lengths’ of these domains, as well as the force required to unfold them can be identified. Due to this it was already possible to show new locations for structural changes in sensory rhodopsin 2 upon light activation. It was also demonstrated, that the conformational answer after light activation varies if another protein is bound to sensory rhodopsin 2 [4]. Further studies on the effect of compatible solutes on bacteriorhodopsin showed a general stabilization of membrane proteins by ectoine [5]. Interactions between two molecules, like TGR5 and a BA could thus also be distinguishable in a similar manner.

Although the AFM allows for us to study membrane proteins in their natural environment, an aqueous solution, membrane proteins, if removed from the cell, have to be stabilized by detergents or a lipid membrane in order to retain their native conformation. We can alternatively either use protein crystals or adsorbe the whole cell to a substrate. However, cells provide a complex system that could hinder the force measurements. Additionally, crystals are not yet available for every membrane protein. To overcome these obstacles artificial lipid bilayer membranes can be used as a suitable approach [6]. In our group we are studying the formation of artificial tethered bilayer lipid membranes (tBLMs) into which a membrane protein can be reconstituted in a defined orientation. Combining the AFM with the reconstitution of a functional membrane protein into a tBLM will allow us to gain structural information on membrane proteins.

## Materials and methods

Single molecule force spectroscopy measurements are performed with the AFM MFP 3D-BIO from Asylum Research using the OMCL-TR400PSA Cantilevers from Olympus.

**Figure 1:**
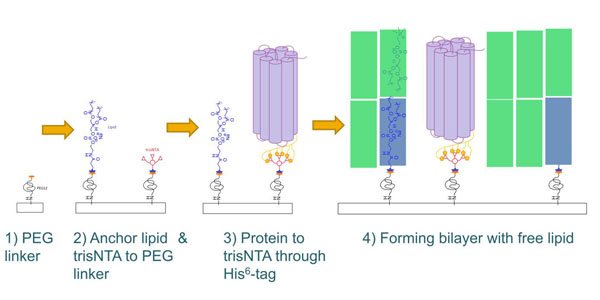
**Coupling Steps & Incorporation of protein into artificial membrane** To reconstitute a membrane protein into a tBLM in a defined orientation we use the following approach. A PEG linker is coupled to a surface functionalized with amino-groups (1). To the PEG linker's free amino-group the anchor lipid, 1,2-dipalmitoyl-sn-glycero-3-phosphoethanol-amine-N-(succinyl) sodium salt and trisNTA are bound (2). Through the proteins His^6^-tag the protein is bound onto the surface with a defined orientation (3). Finally a complete membrane is formed with the free lipid 1,2-dipalmitoyl-sn-glycero-3-phosphocholine (4).

For the force measurements and AFM imaging the protein is reconstituted into a tBLM. To achieve this, a surface is functionalized with polyethylene glycol (PEG) linkers. To these PEG linkers anchor lipids and tris-nitrilotriacetic acid (trisNTA) are bound. A complex is formed between the surface bound trisNTA and the proteins His^6^-tag. This provides the protein in a defined orientation, always with the His^6^-tag containing terminus towards the surface. Detergents in the buffer solution prevent the protein from denaturing while the tBLM is incomplete. Through slow detergent-lipid exchange the tBLM is formed around the protein (Fig. 1).

The specificity of the coupling between trisNTA and His^6^-tag can be studied by reflectometric interference spectroscopy (RIfS).

## Conclusion

In this work we combine the advantage of concentration-controlled protein density and the knowledge about protein orientation due to specific coupling onto a solid support. The support is functionalized with trisNTA, allowing for a membrane proteins His^6^-tag to couple to the surface. The protein is reconstituted into an artificial membrane, a tBLM, by slowly exchanging the detergent solution and forming a bilayer around the protein. This way we can form a tBLM on a solid surface and incorporate membrane proteins with a defined orientation. tBLMs offer a simple and reproducible approach to study membrane proteins in their natural environment. The system can be modified to meet the requirements of different membrane proteins. Protein density and orientation can be controlled by adjustments regarding the surface functionalization. We use this approach to study the membrane protein TGR5 by atomic force microscopy and spectroscopy. However, it could also be used to investigate the structure of other membrane proteins for which a protein crystal could not be obtained, yet.
